# Mitochondrial proteases and their roles in mitophagy in plants, animals, and yeast

**DOI:** 10.1093/pcp/pcaf038

**Published:** 2025-04-23

**Authors:** Kacper Ludwig, Małgorzata Heidorn-Czarna

**Affiliations:** Department of Bioinformatics and Genomics, Faculty of Biotechnology, University of Wrocław, F. Joliot-Curie 14A, Wrocław 50-383, Poland; Department of Molecular Cell Biology, Faculty of Biotechnology, University of Wrocław, F. Joliot-Curie 14A, Wrocław 50-383, Poland; Department of Molecular Cell Biology, Faculty of Biotechnology, University of Wrocław, F. Joliot-Curie 14A, Wrocław 50-383, Poland

**Keywords:** *Arabidopsis thaliana*, *i*-AAA protease, mitochondria, mitochondrial proteases, mitochondrial protein quality control system, mitophagy

## Abstract

Mitochondria play a central role in cellular respiration and other essential metabolic and signaling pathways. To function properly, mitochondria require the maintenance of proteostasis—a balance between protein synthesis and degradation. This balance is achieved through the mitochondrial protein quality control (mtPQC) system, which includes mitochondrial proteases and mitophagy. Mitochondrial proteases ensure proper protein sorting within the mitochondria and maintain proteome homeostasis by degrading unassembled, damaged, or short-lived regulatory proteins. Numerous studies have demonstrated the critical role of mitochondrial proteases in regulating mitophagy—the selective degradation of damaged, aging, or excess mitochondria or their fragments via autophagy. Notably, the rhomboid PARL protease is involved in ubiquitin-dependent PINK1-Parkin mitophagy in mammals, while the *i*-AAA protease Yme1 plays a role in mitophagy in budding yeast. Despite the conservation of core autophagy genes, knowledge about the molecular mechanisms and protein regulators of mitophagy in plants remains limited. In this review, we discuss recent advances in understanding the roles of mitochondrial proteases and mitophagy across plants, animals, and yeast. By comparing these mechanisms across kingdoms, we highlight the potential regulatory function of the plant *i*-AAA mitochondrial protease in controlling mitophagy, providing new insights into mtPQC networks in plants.

## Introduction

Protein homeostasis, or proteostasis, refers to the balance between protein synthesis and degradation. Maintaining proteostasis is crucial for proper cellular function, particularly during growth, development, and stress response. As sessile organisms, plants are particularly vulnerable to environmental changes and biotic stressors, necessitating adaptive responses to fluctuating conditions.

**Figure 1.**
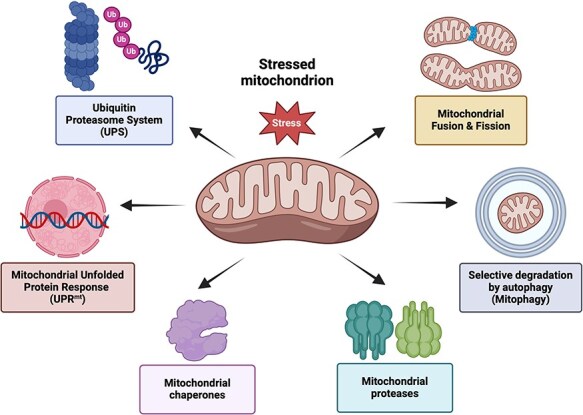
mtPQC mechanisms. Mitochondrial proteostasis is maintained through the activity of mitochondrial chaperones and proteases responsible for protein maturation, repair, refolding, or degradation. When a stress-induced imbalance overwhelms the capacity of the chaperones and proteases, the mitochondrial unfolded protein response (UPR^mt^) is activated, leading to the transcriptional upregulation of nuclear genes encoding the mitochondrial proteins involved in mtPQC. Additionally, the cytosolic UPS is involved in the degradation of the proteins in the outer mitochondrial membrane and intermembrane space. Fusion and fission processes provide organelle-level control of mitochondrial proteostasis by allowing content mixing reduce protein inhomogeneity (fusion) or separating damaged organelles from the mitochondrial network (fission). In cases of terminal mitochondrial damage or specific physiological conditions, mitochondria are directed toward mitophagy–the selective degradation of entire organelles or their fragments via autophagy. The figure was created with BioRender.com.

Mitochondria are complex and dynamic organelles responsible for cellular respiration and essential metabolic processes. Over seven decades of research have revealed that plant mitochondria possess unique features compared with mammalian mitochondria, enabling participation in processes such as photosynthesis, nitrogen and phosphorus metabolism, stress resistance, plant-specific mechanisms of heat production, and programmed cell death (Møller et al. 2021). These diverse activities depend on maintaining mitochondrial proteostasis. Throughout evolution, mitochondria have developed specialized quality control to protect and eliminate defective mitochondrial components or entirely damaged organelles. Mitochondrial proteostasis is maintained through pathways collectively termed the mitochondrial protein quality control (mtPQC) system, which functions at the molecular, organellar, and cellular levels (Fig. 1) (Baker et al. 2011, Fischer et al. 2012).

Mitochondrial proteins are controlled mainly through the activity of evolutionarily conserved mitochondrial chaperones and proteases (Voos 2013). Most mitochondrial proteins are nuclear-encoded, synthesized in the cytosol, and imported post-translationally. Their proper translocation across the outer and inner mitochondrial membranes (OMM and IMM, respectively) and correct folding are facilitated by chaperones, which bind and stabilize unfolded or partially folded polypeptides, thereby preventing aggregation. Many of these chaperones belong to the heat shock protein families, which also aid in disaggregating proteins to restore their correct conformations (Voos 2013). Mitochondrial proteases assist in protein sorting within mitochondrial subcompartments [OMM, IMM, intermembrane space (IMS), and matrix] or in inserting proteins into membranes through limited proteolysis (Ghifari et al. 2019, Heidorn-Czarna et al. 2022). These proteases also maintain proper protein stoichiometries by degrading misfolded, unassembled, or short-lived regulatory proteins in response to growth, developmental, and environmental signals.

When proteostasis becomes imbalanced due to stress, it leads to excess unfolded or misfolded mitochondrial proteins, defective mitochondrial translation and respiration, decreased mitochondrial membrane potential, or impaired import (Tran and Van Aken 2020), thereby activating the mitochondria unfolded protein response (UPR^mt^). This retrograde signaling pathway mediates the upregulation of nuclear genes encoding mitochondrial chaperones, proteases, import proteins, and antioxidant enzymes, adapting proteostasis to cellular or environmental stresses (Zhao et al. 2002).

Another critical quality control system is the ubiquitin-proteasome system (UPS)—a primary protein degradation mechanism in eukaryotic cells—localized in the cytoplasm and nucleus (Ciechanover et al. 1978). Proteins destined for degradation are recognized and modified by the attachment of ubiquitin (Ub) and subsequently degraded by the ATP-dependent 26S proteasome. The UPS degrades the precursors of mitochondrial proteins that accumulate in the cytosol and oxidatively damaged or misfolded mitochondrial proteins located in the OMM or IMS, as well as import intermediates stalled in the pore of the translocase of the outer membrane (TOM) (Heo et al. 2010, Bragoszewski et al. 2015).

Mitochondria are dynamic organelles that undergo cycles of fusion and fission in response to cellular demands (Arimura 2018). Regulation of the mitochondrial network through fusion and fission influences its ultrastructure and mobility and cellular distribution. Fusion and fission also provide an organellar level of control over mitochondrial proteostasis. Fusion allows for the mixing of mitochondrial contents to complement mtDNA mutations and reduce protein quantity inhomogeneity. Conversely, fission separates mitochondria and isolates damaged organelles from the mitochondrial network (Twig and Shirihai 2011). The resulting nonfunctional mitochondria are directed toward mitophagy—the selective degradation of entire organelles or their fragments through autophagy (Nakamura et al. 2021). It is well-established that mitochondrial degradation in lysosomes or vacuoles is a critical quality control mechanism, essential for eliminating damaged and potentially toxic mitochondria or mitochondrial content. The autophagic breakdown of mitochondria is also associated with the developmental transitions observed during tissue differentiation in metazoans (Schweers et al. 2007, Zhang et al. 2021) and plants (Norizuki et al. 2022, Barros et al. 2023). Furthermore, mitophagy has been recognized as a crucial mechanism for maintaining mitochondrial function during senescence in humans (Kelly et al. 2024) and plant cells (Li et al. 2014, Kacprzak and Van Aken 2022).

All mechanisms of mtPQC have evolved into a highly organized and integrated system (Baker et al. 2011, Fischer et al. 2012). Mitochondrial proteases not only maintain mitochondrial proteostasis at the molecular level but also directly influence other mtPQC pathways, including the UPR^mt^, mitochondrial dynamics, and mitophagy. This interconnection highlights the intricate network pathways involved in mtPQC. The role of mitochondrial proteases in the autophagic elimination of mitochondria has been well established in yeast and metazoans. However, despite the conservation of the core autophagy (*ATG*) genes across kingdoms (Yang et al. 2021), the molecular mechanisms and protein regulators of plant mitophagy remained largely unknown until recently.

In this review, we first briefly describe mitochondrial proteases and mitophagy as two crucial mtPQC elements. We then discuss the involvement of the mitochondrial proteolytic system in regulating the autophagic elimination of mitochondria. By comparing these mechanisms across plants, animals, and yeast, we identified the potential regulatory function of plant mitochondrial proteases in controlling mitophagy and provided new insights into the mtPQC network in plants.

## Mitochondrial Proteases

**Figure 2.**
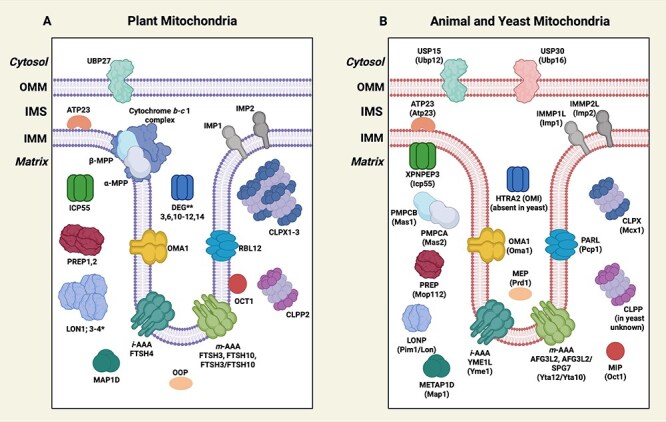
Mitochondrial proteolytic system in plants, animals, and yeast. **(A)** Proteases localized in plant mitochondria, based on *Arabidopsis thaliana*. The schematic shows the ATP-dependent (*i*-AAA, *m*-AAA, LON, CLPX, and CLPP2) and ATP-independent (MPP, OCT1, ICP55, IMP1, IMP2, ATP23, OMA1, RBL12, DEG, OOP, PREP, MAP1D, and UBP27) proteases identified in plant mitochondria. *In *A. thaliana*, the LON1 and LON4 isoforms are dually targeted to the mitochondria and chloroplasts, whereas LON3 is predicted to be a mitochondrial but lacks experimental verification (Ostersetzer et al. 2007). **The exact localization of plant DEG proteases within mitochondria remains uncertain. However, they are hypothesized to be IMS proteins similar to their bacterial homologs, which reside in the periplasmic space, or the mammalian homolog HTRA2, an IMS protein (Schuhmann et al. 2012, Cilenti et al. 2014). (B) Proteases localized in animal and yeast mitochondria. Nomenclature written in capital letters indicates a mammalian protease, in brackets-a yeast protease. OMM, outer mitochondrial membrane; IMS, intermembrane space; and IMM, inner mitochondrial membrane. The proteases ATP23, MAP1D (METAP1D in animals and Map1 in yeast), UBP27 (plants), USP15 and USP30 (animals), as well as Ubp12 and Ubp16 (yeast), are not discussed in the text. ATP23 is a metalloprotease that acts as a processing and quality control protease and a chaperone in OXPHOS biogenesis (Migdal et al. 2017, Heidorn-Czarna et al. 2022). MAP1D removes the N-terminal methionine from nascent proteins (Giglione et al. 2000, Quiros et al. 2015). UBP27, USP15, USP30, Ubp12, and Ubp16 are ubiquitin carboxyl-terminal hydrolases that remove ubiquitin from the target proteins (Cornelissen et al. 2014, Pan et al. 2014b, Quiros et al. 2015). The illustration is based on that described by Heidorn-Czarna et al. (2022). The figure was created with BioRender.com.

Mitochondrial proteases are central components of mtPQC. They are located in all mitochondrial compartments, with the IMM and matrix, harboring a wide array of proteases (Fig. 2). Based on their primary functions, mitochondrial proteases can be categorized into three groups: (i) Processing peptidases: They are involved in limited proteolysis, including the removal of signal sequences from mitochondrial precursor proteins; (ii) Quality control proteases: They degrade damaged, unfolded, or misfolded proteins and prevent the formation of protein aggregates; and (iii) Oligopeptidases: They degrade peptides formed by other proteases. Some mitochondrial proteases exhibit only one specific activity (e.g. limited proteolysis or peptide degradation), whereas others play multiple roles in both protein degradation and processing (Heidorn-Czarna et al. 2022). Mitochondrial proteases also vary in their requirement for ATP. They can be ATP-dependent and ATP-independent mitochondrial proteases (Fig. 2). ATP-dependent proteases primarily function as quality control proteases, whereas ATP-independent proteases mainly serve as processing proteases and oligopeptidases. Numerous comprehensive reviews have described the components of mitochondrial proteolytic systems across different organisms (Quiros et al. 2015, Opalinska and Janska 2018, Deshwal et al. 2020, Ghifari and Murcha 2022, Heidorn-Czarna et al. 2022). Therefore, in this section, we briefly describe the main groups of ATP-dependent and ATP-independent proteases, emphasizing their crucial roles in maintaining mitochondrial proteostasis in plants, yeast, and mammals.

### ATP-dependent proteases

ATP-dependent proteases belong to the AAA+ superfamily (*ATPase associated with various cellular activities*), comprising three families: filamentous temperature-sensitive H (FTSH) proteases (Santos and De Almeida 1975), long form radiation-sensitive (LON) proteases (Howard-Flanders et al. 1964), and caseinolytic proteases (CLP) (Katayama-Fujimura et al. 1987), with some members located in the mitochondria (van Wijk 2015). These proteases possess a proteolytic domain responsible for protein degradation or processing and an AAA domain (also known as a chaperone-like domain), which facilitates the ATP-dependent unfolding of protein substrates, promoting proteolysis. Notably, ATP-dependent proteases can also function as chaperones independently of their proteolytic activity (Lee and Suzuki 2008, Ghifari et al. 2023).

### FTSH proteases

FTSH proteases are metalloproteases containing a conserved zinc-binding motif (HEXXH) within the C-terminal proteolytic domain (Glynn 2017). The AAA and proteolytic domains coexist within the same polypeptide. In mitochondria, FTSH proteases are located in the IMM and include *i*-AAA enzymes (intermembrane space-AAA proteases), which expose their catalytic center to the IMS, and *m*-AAA (matrix-AAA proteases) with a catalytic site directed toward the mitochondrial matrix (Fig. 2). These proteases are essential for maintaining the proteome of the IMM and controlling protein substrates of the OMM and IMS (*i*-AAA) and the matrix (*m*-AAA) (Opalinska and Janska 2018). In yeast and mammals, the *i*-AAA complex forms a homo-hexamer of Yme1 (Yeast Mitochondrial Escape 1) and YME1L (Yeast Mitochondrial Escape 1-Like) subunits, respectively (Thorsness et al. 1993, Weber et al. 1996, Shah et al. 2000). In plants, the only known *i*-AAA protease is FTSH4 (Gibala et al. 2009, Smakowska et al. 2016), although previous studies indicated dual mitochondrial and chloroplast localization of a second *i*-AAA, FTSH11, in aging *A. thaliana* plants (Urantowka et al. 2005). Generally, the *i*-AAA proteases regulate nonassembled, misfolded, oxidized, aggregated, or excess proteins, influencing mitochondrial processes, including oxidative phosphorylation (OXPHOS), protein import, phospholipid metabolism, mitochondrial morphology and dynamics, and signaling (Opalinska and Janska 2018).

The absence of *A. thaliana* FTSH4 protease does not induce visible phenotypic changes under optimal conditions, except during germination (Heidorn-Czarna et al. 2018). However, under stress, *ftsh4* plants display morphological and developmental alterations associated with oxidative stress, accumulation of mitochondrial protein aggregates, phospholipid metabolism imbalance, reduced mitochondrial ATP levels, impaired OXPHOS complex activity, and disrupted mitochondrial morphology characterized by the formation of “giant mitochondria” (Gibala et al. 2009, Hong et al. 2016, Smakowska et al. 2016, Heidorn-Czarna et al. 2018, Maziak et al. 2021). Recent studies demonstrated that both proteolytic and chaperone-like activities of FTSH4 are crucial for preventing mitochondrial protein aggregation during prolonged moderate heat stress (Maziak et al. 2021).

Plant *m*-AAA proteases consist of FTSH3 and FTSH10 subunits forming either homo- or hetero-hexamers (Kolodziejczak et al. 2002). In mammals, *m*-AAA complexes form homo-hexamers of AFG3L2 (ATPase Family Gene 3-Like 2) or hetero-hexamers of AFG3L2 and paraplegin (SPG7) (Koppen et al. 2007). Conversely, in yeast, the *m*-AAA exists solely as a hetero-oligomer comprising the Yta12 and Yta10 subunits (Fig. 2) (Arlt et al. 1996). These proteases primarily function as quality control agents by degrading misfolded, damaged, nonassembled, or incompletely synthesized mitochondrial proteins and are involved in regulating OXPHOS, calcium homeostasis, mitochondrial dynamics, and trafficking (Opalinska and Janska 2018, Ghifari and Murcha 2022). Furthermore, they regulate mitochondrial ribosome biogenesis and protein translation by processing the ribosomal protein MrpL32—the primary role of *m*-AAA in virtually all eukaryotes (Nolden et al. 2005, Kolodziejczak et al. 2018). Contrary to mammals or yeast, plant *m*-AAA complexes are unessential for *A. thaliana* growth and development, as the double *ftsh3/10* mutants exhibit minor developmental changes under optimal growth conditions. However, these mutants display a severe impairment in mitochondrial translation and OXPHOS functionality (Kolodziejczak et al. 2018). Recently, Ghifari et al. (2023) reported that the chaperone-like domain of the *A. thaliana* FTSH3 protease is critical for interacting with the complex I NADH dehydrogenase iron-sulfur protein 7 (PSST). This interaction facilitates the unfolding of complex I matrix arm subunits for degradation.

### LON proteases

LON proteases are highly conserved serine proteases found in bacteria, mitochondria, peroxisomes, and chloroplasts (van Wijk 2015). Each LON subunit contains both the AAA and proteolytic domains and operates as a homo-hexamer or homo-heptamer, depending on the organism (Stahlberg et al. 1999, Cha et al. 2010). In yeast and human mitochondria, LON protease (known as Pim1 in yeast and LONP in humans) plays a crucial role in the folding and assembly of respiratory chain complexes, degradation of oxidatively damaged proteins, gene expression regulation, and maintenance of mtDNA integrity (Suzuki et al. 1994, Van Dyck et al. 1994, Bota & Davis, 2002). Deleting LONP in mammals is lethal, and yeast cells lacking Pim1 exhibit respiratory deficiencies (Van Dyck et al. 1994, Quiros et al. 2014). In *A. thaliana* plants, two mitochondrial matrix isoforms of the LON protease (LON1 and LON4) are dually targeted to mitochondria and chloroplasts (Ostersetzer et al. 2007, Daras et al. 2014). The third isoform, LON3, is also considered a mitochondrial protein (Ostersetzer et al. 2007) even though its localization remains experimentally unverified. LON1 is the primary quality control LON protease in various plant tissues, whereas LON3 and LON4 are expressed exclusively in male and female reproductive organs, respectively (Tsitsekian et al. 2019). Plants lacking LON1 exhibit reduced post-germinative growth, decreased respiratory complex activity, and abnormal mitochondrial morphology (Rigas et al. 2009, Solheim et al. 2012). Recent studies have reported impaired mitochondrial translation, mitochondrial protein aggregate accumulation, and the activation of the mitochondrial unfolded protein response in *A. thaliana lon1* mutants (Song et al. 2024).

### CLP proteases

The CLP proteases family encompasses conserved quality control proteases that degrade misfolded or non-native proteins in prokaryotes, the mitochondrial matrix, and the chloroplast stroma (van Wijk 2015). CLP proteases form a CLPXP system comprising a chaperone-like ATP-dependent polypeptide (CLPX) and a serine protease (CLPP) (Fig. 2). In the absence of CLPX, CLPP degrades proteins nonspecifically, whereas CLPX independently assists in protein folding and unfolding (Lowth et al. 2012, Kardon et al. 2015). Yeast is CLPP deficient, and only a CLPX homolog, a Mcx1 protein devoid of proteolytic activity, has been found in the mitochondria of *Saccharomyces cerevisiae*. In mammals, single isoforms of CLPP and CLPX are found in the mitochondrial matrix, forming a CLPP tetradecamer and two CLPX hexamers (Kang et al. 2005). A list of the identified substrates indicates that the CLP family controls the Krebs cycle, OXPHOS, fatty and amino acid metabolism, and protein translation (Mabanglo et al. 2022). In *A. thaliana* mitochondria, the CLPXP complex consists of the CLPP2 protein and one of the three chaperone-like polypeptides (CLPX1–CLPX3) (Fig. 2). Although relatively little is known about its substrates, structure, and physiological relevance, recent studies revealed that the knockout of the *A. thaliana CLPP2* gene does not impede plant growth and development but disrupts the coordinated expression of nuclear- and mitochondrially encoded subunits of the OXPHOS and ribosomal complexes (Petereit et al. 2020).

### ATP-independent proteases

ATP-independent proteases regulate mitochondrial function and proteostasis through various activities, including: (i) Processing peptidases: They primarily remove the N-terminal presequences in mitochondrial preproteins; (ii) Quality control proteases: They are often involved in more controlled and specific proteolysis compared with ATP-dependent proteases; and (iii) Oligopeptidases: They degrade peptides into amino acids. Below, the most crucial and well-studied ATP-independent proteases are briefly discussed.

### Processing peptidases (MPP, ICP55, OCT1, and IMP)

Different processing proteases localized in the mitochondrial matrix and inner membrane have been identified in plants, yeast, and animals. Among them, the mitochondrial processing peptidase (MPP) is the most widely studied. MPP is a metalloprotease that cleaves N-terminal presequences from mitochondrial proteins after their translocation across the two membranes into the matrix (Sjöling and Glaser 1998). The enzyme also removes mitochondrial targeting sequences that are followed by hydrophobic sorting signals in proteins localized in the IMM and IMS (Burkhart et al. 2015). In yeast and mammals, MPP consists of two structurally related subunits: β-MPP and α-MPP (PMPCB and PMPCA in humans, Mas1 and Mas2 in *S. cerevisiae*), both localized in the mitochondrial matrix (Hawlitschek et al. 1988, Ou et al. 1989). In plants, β-MPP and α-MPP are integrated into the cytochrome *b-c*1 complex of the respiratory chain (Fig. 2) (Braun et al. 1992, Sjöling et al. 1996, Maldonado et al. 2021).

Further processing of proteins cleaved by MPP may be if the new N-terminus contains destabilizing amino acid residues. Intermediate cleavage peptidase 55 (ICP55 in plants, Icp55 in *S. cerevisiae*, and XPNPEP3 in humans) removes a single destabilizing amino acid to stabilize the protein (Vögtle et al. 2009, O’Toole et al. 2010, Carrie et al. 2015, Huang et al. 2015). ICP55 is a highly conserved and typically localized in the mitochondrial matrix of plants (Migdal et al. 2017), whereas in yeast and mammalian mitochondria, it is peripherally attached to the IMM from the matrix site (Fig. 2) (Vögtle et al. 2009). In mammals and yeast, protein substrates containing an arginine (R) residue at the −10 position from the MPP recognition site (the −10 R motif) can be cleaved by the OCT1 protease, which shortens them by eight amino acids (Isaya et al. 1992, Vögtle et al. 2011). Notably, *A. thaliana* OCT1 substrates lack this motif (Carrie et al. 2015). In plants, OCT1 is a membrane-bound enzyme (Migdal et al. 2017), whereas in yeast (Oct1) and mammals (known as MIP, mitochondrial intermediate peptidase), it functions as a mitochondrial matrix protein (Fig. 2) (Isaya et al. 1992, Kalousek et al. 1992).

### Integral inner membrane protease

Integral inner membrane protease (IMP) removes hydrophobic sorting signals that are often present in proteins directed to the IMS or oriented toward the IMS (Nunnari et al. 1993, Mossmann et al. 2012). Two subunits of the IMP protease have been identified in the mitochondria of *S. cerevisiae* (Imp1 and Imp2), humans (IMMP1L and IMMP2L), and *A. thaliana* (IMP1 and IMP2) (Fig. 2) (Nunnari et al. 1993, Migdal et al. 2017). General models for substrate recognition and cleavage motifs of processing peptidases in mammals, yeast, and plants have been extensively reviewed (Ghifari et al. 2019, Heidorn-Czarna et al. 2022).

### Rhomboid proteases

The rhomboid family comprises intramembrane serine proteases that cleave protein substrates within the lipid bilayer through regulated intramembrane proteolysis. Rhomboids are highly conserved proteases found in most living organisms, with some localizing to mitochondria and chloroplasts (Jeyaraju et al. 2013, Adamiec et al. 2017). In *S. cerevisiae* mitochondria, the rhomboid protease Pcp1 (processing of cytochrome *c* peroxidase) cleaves two protein substrates—cytochrome *c* peroxidase (Ccp1) and mitochondrial genome maintenance 1 (Mgm1). Mgm1 is a dynamin GTPase implicated in mitochondrial fusion and cristae formation (Klecker and Westermann 2021). In mammals, the ortholog of yeast Pcp1 is PARL (presenilin-associated rhomboid-like) protease (Fig. 2). However, PARL does not cleave the homolog of Mgm1, an OPA1 (optic atrophy 1) protein (Anand et al. 2014). Instead, it is involved in the highly regulated cleavage of two mitophagy-related proteins, PINK1 (PTEN-induced kinase 1) and PGAM5 (phosphoglycerate mutase 5) (**Table 1**) (Jin et al. 2010, Sekine et al. 2012).

**Table 1.** Proteolytic control of mitophagy: mitochondrial proteases and their substrates in plants, animals, and yeast. *i*-AAA, intermembrane space-AAA proteases; *m*-AAA, matrix-AAA proteases; LON, long form radiation-sensitive protease; CLP, caseinolytic protease; MPP, mitochondrial processing peptidase; OMA1, overlapping with the *m*-AAA protease 1; HtrA2, high-temperature requirement protein A2; 1, 2, 3—despite the lack of identified mitophagy-related substrates for some proteases, evidence suggests their involvement in mitophagy: 1 (Smakowska et al. 2016, Broda et al. 2018), 2 (Wai et al. 2016), 3 (Garcia-Chavez et al., 2024); 4—MPP substrates, for which no specific involvement in mitophagy was shown, are omitted.

	Plants		Yeast		Animals	
Mitochondrial protease (Class)	Protease name	Mitophagy-related substrate	Protease name	Mitophagy-related substrate	Protease name	Mitophagy-related substrate
***ATP-dependent proteases***						
*i*-AAA (metalloprotease)	FTSH4	*unknown* ^1^	Yme1	ATG32 (Wang et al. 2013)	YME1L	*unknown* ^2^
*m*-AAA (metalloprotease)	FTSH3, FTSH10	*unknown*	Yta12	*unknown*	AFG3L2	PINK1 (Greene et al. 2012)
LON (serine protease)	LON1 LON3 LON4	*unknown*	Pim1/Lon	*unknown*	LONP	PINK1 (Thomas et al. 2014)
CLP (serine protease)	CLPP2	*unknown*	*unknown*	*unknown*	CLPP	PINK1 (Greene et al. 2012)
***ATP-independent proteases***						
MPP (metalloprotease)	β-MPP α-MPP	*unknown* ^4^	Mas1, Mas2	*unknown* ^4^	PMPCB, PMPCA	PINK1^4^ (Greene et al. 2012)
Rhomboid protease (serine protease)	RBL12	*unknown*	Pcp1 (Rbd1)	*unknown* ^3^	PARL	PINK1 (Jin et al. 2010); PGAM5 (Sekine et al. 2012)
OMA1 (metalloprotease)	OMA1	*unknown*	Oma1	*unknown*	OMA1	PINK1 (Sekine et al. 2019; Akabane et al. 2023)
HtrA2 (serine protease)	DEG14/ PARK13	*unknown*	*unknown*	*unknown*	HTRA2/ Omi/Park13	Mulan E3 ubiquitin ligase (Cilenti et al. 2014)
Presequence protease (metalloprotease)	PreP1 PreP2	*unknown*	Mop112	*unknown*	PreP	Aβ peptide (Dou and Tan 2023)

The *A. thaliana* genome encodes 19 rhomboid-like proteins, 4 of which are predicted to localize to mitochondria (van Wijk 2015, Adamiec et al. 2017). Of these, only one ortholog of yeast Pcp1, RBL12, has been experimentally characterized in plant mitochondria (Fig. 2) (Kmiec-Wisniewska et al. 2008). Despite the high amino acid sequence, similarity between the *S. cerevisiae* and *A. thaliana* mitochondrial rhomboids, plant RBL12 does not process the yeast substrates, Ccp1 and Mgm1 (Kmiec-Wisniewska et al. 2008). To date, no substrates of plant RBL12 have been identified.

### OMA1

OMA1 (overlapping with the *m*-AAA protease 1) is a highly conserved metalloprotease found in both prokaryotes and eukaryotes, excluding some worms (*Nematoda* and *Trematoda*) and certain flies (*Drosophila*) that have lost the *OMA1* gene (Levytskyy et al. 2017). In mitochondria, OMA1 is localized to the inner membrane, with its zinc-binding motif (HEXXH) positioned on the IMS side, similar to the *i*-AAA proteases (Fig. 2) (Baker et al. 2014). The name OMA1 reflects its ability to replace the *m*-AAA in the proteolytic degradation of the IMM protein Oxa1 (cytochrome OXidase Activity) (Käser et al. 2003). In both yeast and mammals, OMA1 is largely inactive under normal growth conditions. However, it becomes rapidly activated under stress conditions such as heat stress, oxidative stress, or IMM depolarization (Baker et al. 2014, Bohovych et al. 2014, Krakowczyk et al. 2024). In mammals, OMA1 and the *i*-AAA protease jointly regulate the processing of the dynamin-like GTPase OPA1, which regulates mitochondrial dynamics (Anand et al. 2014). Guo et al. (2020) reported that under mitochondrial stress, OMA1 cleaves the IMM-associated DAP3 binding cell death enhancer-1 (DELE1) into a shorter form that translocates to the cytosol, triggering the integrated stress response pathway to maintain cellular and mitochondrial homeostasis. More recently, Krakowczyk et al. (2024) demonstrated that OMA1 mediates the cleavage of mitochondrial precursors arrested within the TOM import channel during membrane depolarization, thereby facilitating the clearance of stalled translocase.

Knowledge about the plant OMA1 protease remains limited. Migdal et al. (2017) were the first to experimentally confirm the presence of OMA1 protease in the *A. thaliana* mitochondria, highlighting its importance in maintaining OXPHOS functionality under both optimal and moderately elevated temperatures. Furthermore, Maziak et al. (2021) demonstrated that OMA1, along with the *i*-AAA protease FTSH4, degrades mitochondrial small heat shock proteins identified in insoluble protein aggregates under prolonged moderate heat stress (30°C) in *A. thaliana* mitochondria.

### HtrA

The ATP-independent HtrA (high-temperature requirement A) family is a group of serine proteases that exhibit dual functionality as both chaperones and proteases. They form trimers and higher-level oligomeric complexes (Hansen and Hilgenfeld 2013). HtrA proteases have been identified in prokaryotes and eukaryotic organelles, including mitochondria, but are notably absent in yeast. In plants and bacteria, the HtrA family is known as DEG proteases (*deg*radation of periplasmic proteins). The *A. thaliana* genome encodes 16 DEG proteins, with DEG6 and DEG16 likely being proteolytically inactive. These proteins are targeted to various cellular compartments, including the chloroplasts, mitochondria, peroxisomes, and nucleus (Schuhmann et al. 2012, Tanz et al. 2014). Using the *A. thaliana* protein subcellular location database (SUBA3) in combination with GFP tagging of DEG protein candidates, Tanz et al. (2014) demonstrated the mitochondrial localization of six DEG proteases in plants, i.e. DEG3 (AT1G65630), DEG6 (AT1G51150), DEG10 (AT5G36950), DEG11 (AT3G16540), DEG12 (AT3G16550), and DEG14 (AT5G27660). Among them, DEG3 and DEG10 were additionally predicted to localize to chloroplasts. However, Huber et al. (2019) reported that DEG10 is exclusively targeted to mitochondria (Fig. 2). According to the updated SUBA5 database (Hooper et al. 2022), DEG3, DEG6, DEG10, DEG11, DEG12, and DEG14 are exclusively mitochondrial proteins. The exact sub-mitochondrial localization of plant DEG proteases remains uncertain. One hypothesis suggests that they are intermembrane space proteins, similar to the mammalian HTRA2 protease, which is an intermembrane space protein and a close homolog of *A. thaliana* DEG14 (Schuhmann et al. 2012, Cilenti et al. 2014). However, given that the chloroplastic DEG2 is localized in the stroma (Schuhmann et al. 2012), it is also possible that some mitochondrial DEGs are localized into the matrix.

Loss of *A. thaliana* DEG10 affects mitochondrial proteostasis and impairs root development, particularly under high temperatures (Huber et al. 2019). Another mitochondrial DEG protease, DEG14 (also known as PARK13), functions as a heat-induced protease that degrades misfolded proteins. DEG14 overexpression enhances the thermotolerance of *A. thaliana* plants (Basak et al. 2014). Additionally, genome-wide mRNA-based analyses have linked DEG14 to a co-expression module associated with thermotolerance and protein unfolding (Majsec et al. 2017). In mammals, HTRA2 (also known as Omi or Park13) is a trimeric protease involved in mtDNA regulation, apoptotic signaling, and mitophagy (Fig. 2) (Quiros et al. 2015). Loss of the *HTRA2* gene results in the accumulation of unfolded mitochondrial proteins, indicating a similar functional role between mammalian HTRA2 and plant DEG14 (Moisoi et al. 2009, Basak et al. 2014).

### Oligopeptidases

The proteolytic products of ATP-dependent proteases, including cleaved mitochondrial presequences and other intramitochondrial protein fragments, are further degraded into amino acids by oligopeptidases present in the mitochondrial matrix and the IMS. The metallopeptidase PREP (comprising PREP1 and PREP2 in *A. thaliana*, Mop112/Cym1 in *S. cerevisiae*, and PREP/PITRM1 in humans) localizes in the mitochondrial matrix, where most of the proteolytic activities related to precursor protein processing and degradation occur. In contrast, the metallopeptidase OOP (OOP in *A. thaliana*, Prd1 in *S. cerevisiae*, and MEP or oligopeptidase M in humans) localizes in the IMS in yeast and mammals, but in the matrix in plants (Fig. 2) (Kmiec et al. 2014). Research suggests that PREP and OOP cooperate in the degradation of shared substrates in *A. thaliana* (Kmiec et al. 2013). Notably, the human homolog of PREP has been directly shown to degrade mitochondrial-localized amyloid-β peptides linked to Alzheimer’s disease (AD), highlighting the crucial role of intra-organellar peptidolysis in the pathogenesis of this disease (Alikhani et al. 2011).

## Mitophagy: An Autophagic Removal of Mitochondria

Cellular organelles are dynamic structures that undergo controlled turnover to recycle their contents, thereby maintaining function and abundance. Autophagy is a process during which organelles or portions of the cytoplasm are captured in double-layered membrane vesicles (autophagosomes) and delivered to lysosomes (in animals) or the vacuole (in yeast and plants) for degradation (Magen et al. 2022). Various cellular structures have been reported to undergo selective autophagy (organellophagy), including protein aggregates (aggrephagy), lysosomes (lysophagy), the endoplasmic reticulum (ER-phagy), peroxisomes (pexophagy), chloroplasts (chlorophagy), and mitochondria (mitophagy) (Stephani and Dagdas 2020). The first documented observation of autophagic elimination of mitochondria dates back almost 70 years when Clark (1957) studied cellular differentiation in the kidneys of newborn mice using an electron microscope. However, mitophagy was not recognized at that time. Plant mitophagy was initially observed in the cotyledon cells of *Vigna mungo* seedlings by Toyooka et al. (2001). The concept of mitophagy, defined as the selective degradation of damaged, aging, excess, or potentially toxic mitochondria or their components through autophagy, was introduced only in 2005, based on studies of the budding yeast *S. cerevisiae* (Lemasters 2005, Priault et al. 2005).

Recognition of the mitochondrial cargo by the autophagy machinery is mediated through interactions between mitochondrial “eat-me” signals and an ubiquitin-like ATG8-family protein conjugated to phosphatidylethanolamine on the autophagosome membrane. The molecular “eat-me” signals are typically divided into receptor- and ubiquitin-mediated signals (Shen et al. 2023). Yeast has a single ATG8 protein (Shpilka et al. 2011), whereas mammals possess six different ATG8 orthologs belonging to the LC3 (microtubule-associated-protein 1 light chain 3) and GABARAP (gamma-aminobutyric acid receptor-associated protein) subfamilies (Onishi et al. 2021). In the model plant *A. thaliana*, the *ATG8* gene family has expanded to nine homologs (Kellner 2017).

### Receptor-mediated mitophagy

#### Protein receptors

Mitophagy receptors are typically transmembrane proteins constitutively residing in the OMM. Upon mitophagy induction, these receptors are activated by post-translational modifications, such as phosphorylation, dephosphorylation, or limited proteolysis. Alternatively, their levels may increase through transcriptional activation or decrease due to degradation. In some cases, the monomeric forms of these receptors must oligomerize, often forming homodimers, to induce mitophagy (Wang et al. 2013, Uoselis et al. 2023). Once activated, receptor proteins recruit the phagophore to enclose the mitochondria by interacting with ATG8 proteins, thereby promoting autophagosome formation. The autophagy machinery recognizes these receptors via conserved ATG8-interacting motifs (AIMs), also known as LC3-interacting regions (Noda et al. 2010).

In yeast species such as *S. cerevisiae* and *Schizosaccharomyces pombe*, the OMM-localized proteins ATG32 and ATG43, respectively, were identified as the first mitophagy receptors (Okamoto et al. 2009, Fukuda et al. 2020). In contrast, mammals possess numerous OMM-localized receptors, including BNIP3 (BCL2 interacting protein 3), NIX (also known as BNIP3L, BCL2 interacting protein 3 like), FUNDC1 (FUN14 domain containing 1), BCL2L13 (BCL2 like 13; a mammalian homolog of yeast ATG32), and FKBP8 (FKBP prolyl isomerase 8) (Chen et al. 1997, Imazu et al. 1999, Shirane and Nakayama 2003, Liu et al. 2012, Murakawa et al. 2015).

Interestingly, plants lack orthologs for most selective mitophagy receptors found in other organisms (Stephani and Dagdas 2020). In *A. thaliana*, the TraB family proteins TRB1 and TRB2 are currently the only identified receptors that interact with ATG8 upon mitophagy induction (Li et al. 2022, Duckney et al. 2024). Previously, *in silico* analyses by Broda et al. (2018) predicted 12 OMM and 36 IMM *A. thaliana* proteins as potential mitophagy receptors containing the AIM motif. Among these were mitochondrial protein import components TOM and TIM (translocases of the inner membrane), voltage-dependent anion channel 2 (VDAC2), elongated mitochondria protein ELM1, hexokinase 1, TRB1 (later confirmed as mitophagy receptor, Li et al. 2022), subunits of the electron transfer chain and ATP synthase, various transporters, and the FTSH4 protease. Further studies are required to verify these predictions.

#### Lipid receptors

The recognition of mitochondrial cargo for degradation through mitophagy can also be mediated by mitochondrial lipids functioning as receptors. Chu et al. (2013) reported that in mammals, mitochondrial damage caused by various stressors leads to the externalization of the inner membrane phospholipid cardiolipin (CL) to the OMM. Once externalized, CL acts as a targeting signal for the recruitment of LC3, a protein that mediates autophagosome formation and cargo recognition. Furthermore, a decrease in the expression of cardiolipin synthase (CLS), the enzyme responsible for cardiolipin synthesis, or phospholipid scramblase-3 (PLS3), which exports CL to the OMM under mitochondrial stress, significantly reduces the recruitment of mitochondria to the phagophore (Chu et al. 2013). In yeast, the loss of cardiolipin impairs mitophagy by decreasing the activation of protein kinase C and high osmolarity glycerol pathways (Shen et al. 2017). However, the link between cardiolipin and mitophagy in *S. cerevisiae* remains unclear. Currently, no direct evidence supports the role of CL in plant mitophagy. However, it has been observed that in *A. thaliana*, the absence of CLS leads to abnormally large mitochondria and higher sensitivity of *cls* protoplasts to programmed cell death effectors (Pineau et al. 2013). Additionally, giant mitochondria and decreased CL levels have been observed in *A. thaliana ftsh4* mutants lacking the mitochondrial FTSH4 protease (Smakowska et al. 2016). Whether the morphological abnormalities and reduced CL content in the *cls* and *ftsh4* mutant plants are linked to defective mitophagy remains to be verified.

Another example of a lipid receptor is C18-ceramide, which selectively targets dysfunctional mitochondria for elimination via mitophagy by directly interacting with LC3 on the mitochondrial membranes in human carcinomas (Sentelle et al. 2012). Currently, there is no evidence supporting the role of ceramides in mitophagy induction in yeasts or plants.

### Ubiquitin-mediated mitophagy

Ubiquitin-mediated mitophagy is a critical mitophagy pathway in metazoans, characterized by the ubiquitination of damaged mitochondria via E3 ubiquitin ligases (Uoselis et al. 2023). It employs autophagy adaptor proteins that recognize polyubiquitinated mitochondrial proteins as mitophagy “eat-me” signals, thereby initiating autophagosome formation through binding to ATG8. The PINK1 kinase/E3 ubiquitin ligase Parkin pathway is the most extensively studied ubiquitin-dependent mitophagy mechanism in humans. This is because PINK1 and Parkin mutations are the major risk factors for familial Parkinson’s disease (Singh and Ganley 2021). PINK1 contains an N-terminal mitochondrial presequence, indicating its mitochondrial matrix localization. However, under steady-state conditions, PINK1 abundance within mitochondria is minimal due to constitutive degradation, as its import into the matrix is directly coupled with degradation (Yamano and Youle 2013). In damaged mitochondria, in which the membrane potential is dissipated, PINK1 import is impaired, causing the full-length PINK1 precursor to accumulate on the OMM in a complex with TOM import translocase (Raimi et al. 2024). Stabilization of PINK1 at the TOM complex on damaged mitochondria is crucial for PINK1 autophosphorylation and subsequent activation. Once activated, PINK1 phosphorylates the ubiquitin-like domain of Parkin (Kondapalli et al. 2012) and ubiquitin molecules already attached to the OMM proteins (Kane et al. 2014). This dual phosphorylation event triggers Parkin activation and its recruitment to the mitochondria, where it intensively polyubiquitinates OMM proteins. The polyubiquitinates process then recruits ubiquitin-binding autophagy adaptor proteins, which target the impaired mitochondria for degradation (Onishi et al. 2021). Several OMM-localized autophagy adaptors have been identified, including OPTN (optineurin), AMBRA1 (activating molecule in Beclin 1 regulated autophagy), SQSTM1/p62 (sequestosome-1), NDP52 (nuclear domain 10 protein 52), TAX1BP1 (Tax1 binding protein 1), and MIRO1 (mitochondrial Rho GTPase 1) (Wang et al. 2011, Heo et al. 2015, Di Rienzo et al. 2022).

In mammalian cells, additional E3 ubiquitin ligases have been shown to drive PINK1-mediated mitophagy independently of Parkin. They include mitochondrial ubiquitin ligase 1 (Mulan/MUL1/MAPL), seven in absentia homolog 1 (SIAH1), and PRB E3 ubiquitin ligase ARIH1 (Rojansky et al. 2016, Szargel et al. 2016, Villa et al. 2017). Further studies are needed to understand how PINK1 activity cooperates with other E3 ubiquitin ligases during mitophagy induction.

Interestingly, the PINK1–Parkin system is absent in yeast, and the dissipation of the mitochondrial membrane potential does not induce mitophagy in *S. cerevisiae* (Abeliovich 2023). However, other ubiquitin-mediated mechanisms may regulate mitophagy in yeast. For instance, Müller et al. (2015) identified the Ubp3–Bre5 deubiquitination complex using a synthetic quantitative array. This complex translocates to the mitochondria and inhibits mitophagy. Similarly, when Parkin is heterologously expressed in yeasts, it stimulates H_2_O_2_-induced protective mitophagy by relocating from the cytosol to the mitochondria in response to oxidative stress (Pereira et al. 2015). These findings strongly suggest that some components of ubiquitin-mediated mitophagy, including mechanisms governing ubiquitin ligase recruitment and mitophagy adaptors, are conserved in yeast.

Plants lack homologs of the PINK1–Parkin pathway. However, recent research has presented evidence supporting depolarization-induced mitophagy in *A. thaliana* (Ma et al. 2021, Kacprzak and Van Aken 2022). This process involves a protein called Friendly Mitochondria (FRIENDLY; FMT) (Ma et al. 2021). FMT is a member of the highly conserved CLUSTERED MITOCHONDRIA (CLU) protein superfamily, which is necessary for proper mitochondrial distribution and preventing mitochondrial clustering within the cell (Fields et al. 1998, Cox and Spradling 2009, El Zawily et al. 2014). In *Drosophila melanogaster*, the Clu protein interacts with PINK1 and Parkin and is required for PINK1–Parkin mitophagy upon mitochondrial depolarization (Sen et al. 2015, Wang et al. 2016). In contrast, in *A. thaliana*, FMT is predominantly localized in the cytosol, where it functions to maintain normal mitochondrial distribution, motility, and fusion (El Zawily et al. 2014). A fraction of FMT binds mRNA and associates with cytosolic ribosomes on the mitochondrial surface (Hemono et al. 2022). Ma et al. (2021) demonstrated that in the *A. thaliana fmt* mutants, treatment with uncouplers that disrupt the mitochondrial proton gradient reduces mitophagy levels and results in the accumulation of abnormal autophagosomes. During dark-induced senescence, mitochondrial association of FMT, decreased mitophagy, and increased cluster size and number of the *fmt* mutants have also been observed (Kacprzak and Van Aken 2023). These findings suggest that FMT is essential for the elimination of dysfunctional mitochondria, although it remains speculative whether FMT acts directly as a mitophagy receptor. It has been proposed that FMT facilitates efficient mitophagy by preventing mitochondrial clustering, thereby allowing mitochondria to be more effectively engulfed by autophagosomes (Kacprzak and Van Aken 2023).

To our knowledge, no evidence supports the involvement of E3 ubiquitin ligases in plant mitophagy. Homologs of most ubiquitin-dependent mitophagy adaptors are also absent in plants (Stephani and Dagdas 2020). Although under debate, the observation of the E3 ubiquitin ligase SP1 (Suppressor of *ppi1*)—a counterpart of the mammalian Mulan/MUL1/MAPL ligase—in the mitochondrial membranes of *A. thaliana* (Pan and Hu 2017) suggests its possible role in plant mitophagy regulation. Interestingly, treatment with uncouplers induces both mitophagy and mitochondrial protein ubiquitination in *A. thaliana* (Ma et al. 2021). Further research is required to determine whether these processes are functionally linked.

The above-mentioned “eat-me” signals act on the surface of the OMM. Interestingly, an IMM protein, prohibitin 2 (PHB2), has also been identified as a mitophagy receptor in the PINK1–Parkin pathway. PHB2 directly binds to LC3, promoting mitophagy upon mitochondrial depolarization and rupture of the OMM (Wei et al. 2017). The reasons behind the necessary OMM rupture and the involvement of an IMM-localized protein in autophagosome formation during PINK1–Parkin-mediated mitophagy remain unclear. Additionally, the mechanism underlying OMM rupture requires further investigation, although it is known to involve proteasome activity (Wei et al. 2017). Recently, prohibitins (PHBs) Phb1 and Phb2 were identified as mitophagy receptors in yeast, in which their interaction with ATG8 has been found necessary for the selective autophagic degradation of mitochondria (Garcia-Chávez et al. 2024). Moreover, Phb1 and Pb2 influence the processing and stability of the ATG32 receptor. In *A. thaliana*, PHBs were not identified among the putative mitochondrial ATG8-interacting proteins (Broda et al. 2018). However, their role in plant mitophagy should not be dismissed, given their critical roles in mitochondrial biogenesis and function (Ahn et al. 2006, Van Aken et al. 2007).

## Direct Contribution of Diverse Mitochondrial Proteases to Mitophagy

**Figure 3.**
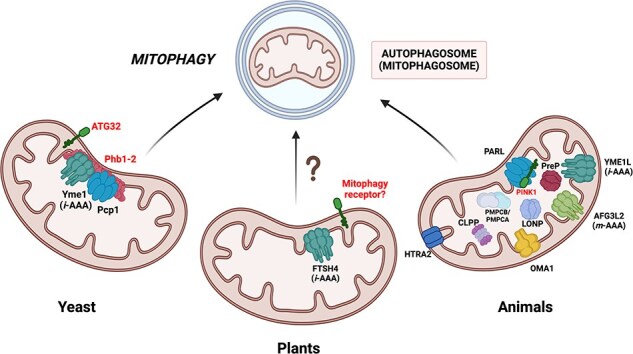
Mitochondrial proteases involved in mitophagy in plants, animals, and yeast. The mitophagy-mediating proteins (PINK1 in mammals; ATG32, Phb1, and Phb2 in yeast) known to be regulated by mitochondrial proteases are highlighted in red. The *i*-AAA protease FTSH4 is proposed to play a role in proteolytic control of plant mitophagy. Details regarding specific proteases and their roles in mitophagy are discussed in the main text. The figure was created with BioRender.com.

Understanding the molecular mechanisms that drive cells to eliminate mitochondria via mitophagy is crucial for elucidating its physiological functions and its role in disease. Because mitophagy and mitochondrial proteostasis are integral components of the mtPQC system, mitochondrial proteases are expected to be essential regulators of mitophagy. Over the past 15 years, numerous studies have provided substantial evidence of the crucial role of mitochondrial proteases in mitophagy regulation (**Table 1**, Fig. 3). One of the most recognized cases is the involvement of rhomboid PARL in the ubiquitin-dependent PINK1–Parkin mitophagy pathway in mammals. Early studies revealed that, in healthy mitochondria, the import of the PINK1 precursor through the mitochondrial membranes is coupled with the cleavage of its mitochondrial presequence by the MPP, generating a 60-kDa form of PINK1 (Greene et al. 2012). Further limited proteolysis of PINK1 is catalyzed by the PARL protease, which processes PINK1 at the transmembrane segment, yielding a shorter 52-kDa protein. This PARL-mediated cleavage exposes an N-terminal phenylalanine, which acts as a destabilizing signal according to the N-degron pathway, marking the protein for ubiquitination and subsequent degradation by the 26S proteasome (Yamano and Youle 2013). The cleaved PINK1 is then retranslocated to the cytosol through an unknown mechanism, in which it undergoes ubiquitination and degradation (Yamano and Youle 2013).

However, PARL is not the only mitochondrial protease involved in the PINK–Parkin pathway. Other proteases may have partially redundant functions. Greene et al. (2012) reported that the 52-kDa PINK1 protein form can also be generated by the *m*-AAA protease AFG3L2 and reduced expression of either PARL or AFG3L2 significantly increased levels of the MPP-processed PINK1 within mitochondria. Notably, PINK1 cleavage by CLPP has also been observed, although it was less efficient than cleavage by PARL or *m*-AAA (Greene et al. 2012). Interestingly, Thomas et al. (2014) showed that in *D. melanogaster*, processed PINK1 is directed to the cytoplasm and the mitochondrial matrix, where it is degraded by the LON protease. More recent studies have demonstrated that upon dissipation of mitochondrial potential, PINK1, which fails to accumulate on depolarized mitochondria, is regulated by the OMA1 protease (Sekine et al. 2019). Additionally, the Tim23 subunit of the TIM import translocase protects PINK1 from OMA1-mediated degradation (**Table 1**, Fig. 3) (Akabane et al. 2023).

In contrast to the PARL-mediated processing of PINK1, which occurs in healthy mitochondria, cleavage of another mitophagy-related protein, the IMM-localized phosphatase PGAM5, by PARL occurs in response to the mitochondrial membrane potential loss (**Table 1**) (Sekine et al. 2012). Consequently, PGAM5 dephosphorylates and thus activates the FUNDC1 receptor, enabling its interaction with ATG8 at damaged mitochondria (Chen et al. 2014, Uoselis et al. 2023).

The mammalian IMS-localized HTRA2 protease has emerged as a significant regulator of both apoptosis and mitophagy. During apoptosis induction, HTRA2 is released into the cytoplasm, where it participates in cell death. Under normal conditions, it remains confined to the IMS, exhibiting pro-survival functions associated with maintaining mitochondrial proteostasis (Chao et al. 2008). Cilenti et al. (2014) identified the mitochondrial Mulan E3 ubiquitin ligase (MUL1/MAPL) as a specific substrate of HTRA2 (**Table 1**). Under normal conditions or during exposure to various moderate stressors, HTRA2 degrades Mulan. However, deregulations leading to Mulan accumulation—such as *HTRA2* gene knockout—cause mitochondrial dysfunction and trigger mitophagy. The authors proposed that the Mulan/HTRA2 pathway acts as a Parkin-independent mechanism of mitophagy in mammals (Fig. 3). Currently, there is no information regarding the role of the *A. thaliana* counterpart of the mammalian Mulan E3 ubiquitin ligase, known as the E3 ubiquitin ligase SP1 (Pan and Hu 2017), in plant mitophagy. Additionally, the proteolytic regulation of the DEG14 (PARK13) protease in this process remains unexplored.

It is well established that amyloid-β (Aβ) peptides accumulate in the mitochondria of patients with AD, and the oligopeptidase PreP is responsible for degrading these peptides (Alikhani et al. 2011). Recently, Dou and Tan (2023) demonstrated that excess Aβ peptides in mammalian mitochondria can impair organelle function and increase mitophagy. Their findings also showed that by degrading Aβ peptides, PreP protects mitochondria from damage by reducing mitophagy levels (**Table 1**, Fig. 3).

## Multi-Level Involvement of the *i*-AAA Protease in Mitophagy

In contrast to the direct interactions of the aforementioned proteases with mitophagy-related substrate proteins, an indirect contribution of another mammalian mitochondrial protease, the *i*-AAA protease YME1L, to mitophagy has been demonstrated by Wai et al. (2016). They showed that PINK1 and PGAM5 cleavage by PARL occurs within a large proteolytic hub known as SPY. This hub is composed of the YME1L protease, stomatin-like protein 2 (SLP2), and PARL itself, with SLP2 acting as a scaffold protein anchoring both proteases to the membrane. Within the SPY hub, YME1L facilitates the processing of PINK1 by PARL and modulates its activity toward PGAM5 (Wai et al. 2016). However, the precise mechanism underlying the regulation of the PARL proteolytic activity by YME1L remains unelucidated.

Unlike mammals, where YME1L has an indirect role in mitophagy, the *i*-AAA protease Yme1 in the yeast *S. cerevisiae* functions as a direct regulator of mitophagy. Early research revealed that *S. cerevisiae* cells lacking Yme1 display enlarged and swollen mitochondria (Campbell et al. 1994). Later, Wang et al. (2013) revealed that Yme1 acts as a processing protease for the OMM mitophagy receptor ATG32 (**Table 1**, Fig. 3). Upon mitophagy induction, Yme1 cleaves the C-terminal region of ATG32 protruding into the IMS, thereby enhancing the interaction of ATG32 with the adaptor protein ATG11. This interaction facilitates mitochondrial recruitment and mitophagosome formation. Blocking this processing significantly impairs mitophagy (Wang et al. 2013).

However, the influence of Yme1 on mitophagy appears to be more complex than expected. Under some experimental conditions, the deletion of Yme1 actually promotes mitophagy rather than inhibiting it (Welter et al. 2013). Over 30 years ago, Yme1 inactivation was found to increase mtDNA leakage to the nucleus, seemingly dependent on the stimulated vacuolar digestion of mitochondria (Thorsness et al. 1993). Increased mitophagy has also been reported in mutants lacking the Yme1 homolog in other fungal species, such as *S. pombe* (Vega et al. 2022) and the filamentous fungus *Podospora anserina* (Löser et al. 2021). However, it should be noted that these species do not possess a homolog of the mitophagy receptor ATG32 (Fukuda et al. 2020, Löser et al. 2021). Therefore, it can be hypothesized that in *S. cerevisiae*, Yme1 can influence mitophagy both directly—through ATG32 activation (Wang et al. 2013)—and indirectly by degrading dysfunctional mitochondrial proteins, thereby mitigating mitochondrial stress (Welter et al. 2013). Depending on which function prevails under specific conditions, Yme1 mutation can either inhibit or stimulate mitophagy.

Furthermore, recent research has revealed that ATG32 processing is not exclusively activating but, if excessive, can also destabilize ATG32 (Garcia-Chávez et al. 2024). Yeast prohibitins, in addition to functioning as receptors, can regulate Yme1 activity, as their mutations result in an abnormal and destabilizing increase in ATG32 cleavage. Interestingly, this effect is partially suppressed by an additional mutation in Pcp1, the yeast ortholog of the PARL protease. It is unlikely that Pcp1 directly processes ATG32, as the rhomboid protease cleavage sites are located within the IMM. This suggests that SPY-like complexes can exist in both mammals and yeast, possibly with prohibitins acting as scaffolds instead of stomatin-like proteins and with Pcp1 regulating Yme1 activity, opposite to mammals (Wai et al. 2016).

*S. cerevisiae* cells lacking tafazzin (*taz1*Δ) serve as a yeast model of Barth syndrome in humans, a lethal cardiomyopathy characterized by decreased CL levels in mitochondria (Gaspard and McMaster 2015). Reportedly, Yme1 protease deletion exacerbates the phenotype of the *taz1*Δ mutant. Yme1 degrades Ups1 and Ups2 proteins, which regulate CL biosynthesis (Potting et al. 2010). Nonetheless, the Yme1 mutation did not influence the CL levels in *taz1*Δ yeasts. Instead, Yme1 inactivation in *taz1*Δ cells caused severe defects in mitochondrial morphology, manifested as giant mitochondria lacking cristae, and led to a substantial impairment of mitophagy, stronger than in the *yme1*Δ mutant. Enlarged mitochondria with markedly altered cristae have also been observed in human embryonic kidney cells with stable YME1L deletion (Stiburek et al. 2012) or YME1L deficiency (Anand et al. 2014), as well as in the *D. melanogaster dYME1L^del^* knockout mutant (Qi et al. 2016).

There is no close homolog of ATG32 in *A. thaliana* (Broda et al. 2018). Nonetheless, abnormally large mitochondria, alongside normal oval ones, were observed in *A. thaliana ftsh4* plants, which lack the *i*-AAA protease FTSH4 (Smakowska et al. 2016). Under optimal growth conditions (long-day photoperiod, LD; 22°C), giant mitochondria were rare, but their number and size increased under moderate heat stress (LD; 30°C). Additionally, in aging *ftsh4* mutant plants grown under restricted light conditions, mitochondria exhibited poorly developed cristae (Gibala et al. 2009). Unlike findings in *taz1*Δ yeast cells, these morphological alterations may be linked to phospholipid metabolism, as *ftsh4* mutants displayed decreased CL levels in mitochondrial membranes, especially at 30°C (Smakowska et al. 2016). Supporting this notion, a mutation in CL synthase also causes mitochondrial enlargement in *A. thaliana* (Pan et al. 2014a).

Furthermore, Zhang et al. (2017) revealed that the loss of FTSH4 in *A. thaliana* significantly increases the number of dead cells and autophagosomes in *ftsh4* mutant plants, suggesting that FTSH4 is likely involved in the ATG5- and ATG8-mediated autophagy pathway. However, it remains unclear whether the autophagosomes observed are mitochondria-specific. Interestingly, an *in silico* analysis of the AIM in *A. thaliana* mitochondrial proteins revealed the presence of AIM within FTSH4, indicating a putative role for the plant *i*-AAA protease as a receptor protein in mitophagosome formation (Broda et al. 2018). The localization of FTSH4 in IMM suggests that the protease functions as a mitophagy receptor when severe mitochondrial damage, accompanied by OMM rupture, occurs (Wei et al. 2017). It is also possible that in plants, similar to yeast, the FTSH4 protease participates in mitophagy through the proteolytic cleavage of a specific OMM mitophagy receptor that interacts with the ATG8 protein conjugated to the autophagosome membrane. However, further research is required to determine precisely how FTSH4 affects plant mitophagy.

Collectively, these studies suggest that the *i*-AAA protease is associated with autophagy and mitophagy and offers insights into its role in maintaining mitochondrial morphology. To date, only in the budding yeast *S. cerevisiae* has the direct link between the Yme1 protease and mitophagy been demonstrated (Wang et al. 2013). Although the molecular mechanisms underlying the formation of morphologically altered mitochondria in *i*-AAA-deficient cells may differ across kingdoms, ineffective mitophagy in *i*-AAA-deficient cells could result from a common cause: the size of giant mitochondria may hinder the autophagic machinery from efficiently engulfing the enlarged organelles. Over time, the incomplete elimination of defective mitochondria in *i*-AAA mutant cells may result in the accumulation of cellular damage, leading to functional decline and cell death.

## Conclusions and Open Questions

To date, most research on plant mitophagy has focused on identifying mitophagy triggers rather than elucidating the underlying mechanisms and regulatory pathways. Various conditions have been shown to trigger plant mitophagy, including mitochondrial depolarization (Ma et al. 2021, Kacprzak and Van Aken 2022), UV-B damage (Dündar et al. 2020), natural senescence (Li et al. 2014, Kacprzak and Van Aken 2022), and carbon starvation (Kacprzak and Van Aken 2022). Recently, it has been demonstrated that during spermiogenesis in *Marchantia polymorpha*, unnecessary mitochondria are eliminated via mitophagy, preceding the autophagic degradation of other cellular components (Norizuki et al. 2022). A similar role of mitophagy in sperm differentiation is also expected in *Physcomitrium patens* based on preliminary results (Sanchez-Vera et al. 2017). In growing *A. thaliana* pollen tubes, which exhibit intensive mitochondrial metabolism, mitochondria are sometimes found within autophagosomes, suggesting that autophagy is essential for maintaining tube growth. However, the occurrence of specific mitophagy in pollen tubes has only been demonstrated following uncoupler treatment (Yan et al. 2023).

These studies emphasize the critical physiological functions of mitophagy in plant cells, particularly in stress resistance. The plant response to various environmental factors is intensively studied, as insights gained can lead to novel biotechnological applications that minimize crop failures associated with environmental stresses (Mantri et al. 2012). This is especially important in the age of global warming and its associated extreme weather events. However, practical applications of mitophagy-related knowledge remain limited due to a lack of data on the molecular mechanisms of mitophagy and their regulation. This highlights the importance of recent studies that identified the first specific regulators of plant mitophagy—the FMT protein and TRB1-2 receptors (Ma et al. 2021, Li et al. 2022, Kacprzak and Van Aken 2023). Investigating their interaction partners or applying similar methodologies to other candidate mitophagy regulators, such as plant prohibitins and the *i*-AAA protease, may soon lead to the identification of new proteins involved in plant mitophagy. Moreover, it would be interesting to determine whether CL is involved in plant mitophagy, as in mammals (Chu et al. 2013).

Compared with the well-studied case of PINK1 cleavage by the mammalian PARL protease and the established involvement of numerous proteases in regulating mitophagy in yeast and animals, little is known about the interconnection between plant mitochondrial proteolysis and mitophagy. Although preliminary and requiring further verification, recent discoveries regarding the potential roles of FTSH4 in mitophagy (Smakowska et al. 2016, Zhang et al. 2017, Broda et al. 2018) offer promising new research avenues in this field. It also remains to be determined whether the homologs of various proteases involved in animal and yeast mitophagy—such as RBL12 (PARL homolog), DEG14 (HTRA2 homolog), OMA1, LON1, FTSH3 and FTSH10 (*m*-AAA proteases), MPP, and PREP1-2—are also involved in regulating plant mitophagy.

## Data Availability

No new datasets were generated or analyzed in this study.
